# A longitudinal study of tobacco use among American Indian and Alaska Native tribal college students

**DOI:** 10.1186/1471-2458-10-617

**Published:** 2010-10-18

**Authors:** Babalola Faseru, Christine M Daley, Byron Gajewski, Christina M Pacheco, Won S Choi

**Affiliations:** 1 Department of Preventive Medicine and Public Health, University of Kansas Medical Center, Kansas City, KS, USA; 2 Center for American Indian Community Health, University of Kansas Medical Center, Kansas City, KS, USA; 3 Department of Biostatistics, University of Kansas Medical Center, Kansas City, KS, USA

## Abstract

**Background:**

American Indians (AI) have the highest smoking rates of any ethnic group in the US (40.8%), followed most closely by African Americans (24.3%) and European Americans (23.6%). AI smokers also have more difficulty quitting smoking compared to other ethnic groups, evidenced by their significantly lower quit ratios, and are among the least successful in maintaining long term abstinence. While health disparities like these have existed for years among AI, the epidemiology of smoking and nicotine dependence has not been optimally described among this underserved population.

Our overarching hypothesis is that the susceptibility of AI to cigarette smoking and nicotine dependence and its consequences has both an underlying nicotine metabolism component as well as psychosocial, cultural, and environment causes. We are well-positioned to explore this issue for the first time in this population. Our objective is to establish a cohort of AI tribal college/university students to determine the predictors of smoking initiation (non-use to experimentation), progression (experimentation to established use), and cessation (established use to cessation). Much of what is known about the process of smoking initiation and progression comes from quantitative studies with non-Native populations. Information related to smoking use among AI tribal college/university (TCU) students is entirely unknown and critically needs further investigation. This study will be the first of its kind among AI college students who are at the highest risk among all ethnic groups for tobacco dependence.

**Methods/design:**

First year students at Haskell Indian Nations University in Kansas will be recruited over four consecutive years and will be surveyed annually and repeatedly through year 5 of the study. We will use both longitudinal quantitative surveys and qualitative focus group methods to examine key measures and determinants of initiation and use among this high risk group.

## Background

Cigarette smoking is the number one cause of preventable death among American Indians and Alaska Natives (AI/AN) [1,2]. The mortality rate among AI/AN due to tobacco use is double that of others in the United States [3]. Two out of every five AI/AN and one out of every two AI/AN smokers will die from tobacco-related diseases. Cancer is the second leading cause of death among AI and is the leading cause of death among AN [4]. Lung cancer is the leading cause of cancer death for AI/AN men and women. There is variability in lung and bronchus cancer mortality among Native Americans by region (high in the Northern Plains (93.6 per 100,000) and Alaska (70.5 per 100,000) and lower in the Southwest (12.9 per 100,000) and East (40.2 per 100,000) due to regional differences in smoking prevalence rates. The decrease in cancer mortality in the US starting in the mid-1990s did not extend to AI/AN who experienced increased mortality when comparing 1990-1995 to 1996-2001. Lung cancer mortality during this time period increased from 38.75 to 41.06/100,000. Recent triangulation of cancer data shows much racial miscoding and suggests that incidence and mortality rates for all cancers in AI/AN may be underestimated by as much as 100% [5].

The overall leading cause of death for both male and female AI/AN is cardiovascular disease (CVD), 158.2 and 113.2 per 100,000 respectively. Tobacco is a key risk factor [6,7]. The proportion of premature deaths attributed to CVD is greatest among AI/ANs (36.0%, compared to 14.7% for European Americans) [8]. The AI/AN CVD rate for the three-year period of 1996-98 was 20% greater than the US all-races rate (157.1 compared to 130.5). These CVD mortality rates for AI/AN likely underestimate true rates. A recent study on racial misclassification and disparities in CVD found that the total CVD mortality for AI/AN was higher than in the rest of the nation and may have been higher for more than a decade [9].

Among men and women combined, the prevalence of smoking is highest among AI/ANs compared with European Americans or any other racial/ethnic group. Smoking rates among AI/AN smokers vary by region and are highest in the Northern Plains 44.1%, and lowest in the Southwest 21.2% [10,1]. Among the 18-24 year age group, national data show that AI/AN have the highest current smoking prevalence at 46% [11]. AI/AN smokers also have more difficulty quitting smoking compared to other ethnic groups [3], evidenced by their significantly lower quit ratios [12,13], and are among the least successful in maintaining long term abstinence. In 2000, 70% of AI/AN smokers said they wanted to quit, and 41% made a quit attempt of at least one day, but only 5% succeeded in quitting for three months [14]. In 2000, 41% of all AI/AN who had ever smoked reported that they had successfully quit, compared with 51% of European Americans [3]. The Strong Heart Study, a longitudinal study of cardiovascular disease risk in AI/AN, found several factors were significantly related to cessation among older AI/AN, including a history of diabetes, beginning to smoke at age 17 or older, being a smoker for fewer years, smoking fewer than six cigarettes per day, not smoking every day, and being 65-74 years old. Both smoking and use of smokeless tobacco are highest in the AI/AN population. One of the contributing factors is the influence of the tobacco industry and their marketing practices. To build its image and credibility in Native communities, the tobacco industry targets AI/ANs by funding cultural events such as pow-wows and rodeos as well as providing funds directly to AI/AN TCUs. In addition, the tobacco industry commonly uses cultural symbols and designs in their marketing practices to specifically target AI/ANs.

There are approximately 25,000 students enrolled in TCUs in the US, and the number of students enrolling has increased over the past decade [15]. During the 1990s, enrollment of AI/AN students at TCUs increased by 62%. In comparison, AI/AN student enrollment increased by 36% at mainstream colleges over the same time period [15].

Data from the Monitoring the Future Survey show that racial/ethnic smoking prevalence is highest among AI/AN high school seniors (males 41.1%, females 39.4%) [10]. In a national study of US high school seniors, AI/ANs reported the highest levels of tobacco, alcohol and illicit drug usage across all ethnic groups between 1996 and 2000. In a 2003 survey among South Dakota high school students, more than 56% of AI/AN youth replied affirmatively when asked if they have ever tried smoking [16]. In the same survey more than 28% were current smokers. In a study of 906 public school AI/ANs between 6^th ^and 8^th ^grade, 45% reported current usage of some tobacco product, with 79% reported having tried a tobacco product. Current use of tobacco products was categorized as cigarettes 39%, smokeless tobacco (SLT) 15%, and cigars 12% [17]. Despite high prevalence rates of AI/AN adolescents, there is little research on initiation patterns and possible socio-cultural and environmental differences that may contribute to smoking initiation.

Information on prevalence rates of smoking among AI/AN college students is limited. With increasing numbers of AI/AN adolescents attending or expecting to attend college and the high prevalence of smoking in this group, it is important to examine behaviors and risk factors associated with smoking. The TCU environment is the perfect place to begin to learn this information due to the high percentage of AI/AN students and the rapidly increasing enrollment. Once we understand smoking initiation, progression and cessation and the risk factors in this group, we can begin to target cessation and prevention programs specifically to this high risk population.

Such factors relating to tobacco use and nicotine dependence and metabolism may be different between AI/ANs and the general US population, and even among AI/AN subgroups [18]. In the non-AI/AN population, it has been shown that cotinine concentration is associated with age, years of smoking, cigarettes per day, and inhalation depth. Most of this information is based on studies of non-AI/AN smokers. It is important to examine the psychosocial factors as well as the smoking patterns with cotinine concentrations in the AI/AN population because such relationships are important when developing effective cessation interventions.

Our overarching hypothesis is that the susceptibility of AI/ANs to smoking and nicotine dependence and its consequences has both an underlying nicotine metabolism component as well as psychosocial, cultural and environmental causes. This study will be the first of its kind among AI/AN college students who are at the highest risk among all ethnic groups for tobacco dependence. Our objective is to establish a cohort of AI/AN TCU students to determine the predictors of smoking initiation (non-use to experimentation) and progression (experimentation to established use). Our second objective is to use qualitative methods to gather information that will be used to inform the future design and implementation of prevention and cessation programs for smoking. We will also examine the nicotine metabolism in current smokers in this population. The study was approved by The Haskell Indian Nations University Institutional Review Board and the University of Kansas Medical Center Human Subjects Review Committee.

### Study objectives

We will use both quantitative (longitudinal surveys) and qualitative (focus groups) methods to address the following study aims:

#### Study Aim 1

To examine the natural history of cigarette smoking among American Indian and Alaska Native tribal college/university students.

a) To estimate the prevalence of cigarette smoking in this population.

b) To determine the rates of smoking uptake among these students.

c) To determine the rates of cessation and quit attempts in this population.

#### Study Aim 2

To examine factors (e.g., role of traditional tobacco use, tobacco marketing, etc) associated with cigarette smoking and cessation among American Indian and Alaska Native tribal college/university students.

a) To determine the influence of traditional tobacco use on cigarette smoking.

b) To examine the influence of personal and environmental factors, such as on-campus tobacco marketing, on the uptake of smoking.

#### Study Aim 3

**To examine the nicotine metabolism in American Indian and Alaska Native tribal college/university students who are current smokers**.

a) To characterize the rate of nicotine metabolism in current smokers.

#### Study Aim 4

**To collect information about what American Indian and Alaska Native college students would like to see in a smoking cessation program designed specifically for them**.

Although recent studies have addressed tobacco use among college students, none has examined cigarette smoking among AI/AN TCU students through a longitudinal cohort study. Compared with cigarette smoking in the non-Indian population, we know much less about the factors influencing smoking uptake, progression and cessation in this population. The proportion of AI/ANs who attend college/university has been increasing over the past decade, while cigarette smoking has also increased and remained high in this population over the same period. The ability to conduct in-depth focus groups with an AI/AN student population will provide an opportunity to gather information that can be used to inform the design and delivery of culturally tailored interventions for smoking prevention and cessation. This study will also be the first to collect saliva from current smokers to examine the issues related to nicotine metabolism in the AI/AN population because so little is known about it in this population. Much of what is known about the process of smoking initiation and progression comes from quantitative studies with non-Native populations [19]. Information related to cigarette smoking among AI/AN TCU students is entirely unknown and critically needs further investigation.

Several prevention based smoking interventions have been tested among adolescent and grade school AI/AN populations with limited success [20]. Programs that have had limited successes have considered the two cultures in which many Native youth live: AI/AN culture and the dominant non-Native culture. These programs have been guided by the theory that youth who understand both cultures may select the most advantageous behavior and cultural ideology of both cultures versus those who only identify with one. This bicultural competency allows for enhanced decision making, problem solving and media analysis skills. The search for long-lasting, effective programs that reduce smoking prevalence will be advanced by the aims outlined in this study [20]. A few programs have been designed for smoking cessation among AI/ANs and may provide insight into smoking intervention. The "It's Your Life - It's Our Future" project in Northern California used messages related to cultural identity, responsibility to family and tribe and respect for tobacco products [13]. The program was tailored to the California AI/AN community and had a 5.7% quit rate at 18-month follow-up for the intervention group versus a 3.1% quit rate for the control group. Currently, there are no effective programs that have been developed for AI/AN youth and young adults.

It is clear from recent studies that tobacco use, especially smoking, has been and still remains a major problem among AI/AN. Due to the high prevalence of smoking among AI/AN, the National Cancer Institute has urged that this population be targeted for prevention and cessation activities. The data relevant to tobacco use during college suggest that this is a time of transition in terms of tobacco use behavior. While some may quit smoking during college, others will experiment and become frequent smokers, and others will firmly establish their addiction. Developing a better understanding of ethnic differences in the collegiate setting of smoking initiation, progression and cessation may offer insights into opportunities for disrupting the transition to addiction. Understanding the recreational value of smoking and cultural conflicts between AI/ANs and the larger society will be essential. The resultant findings could identify culturally sensitive and effective intervention opportunities to reduce the burden of smoking in this population.

## Methods and Design

### Overview

The primary aim of this study is to examine the natural history of smoking among AI/AN TCU students. We will conduct an observational cohort study to ascertain measures of use and examine risk factors for smoking. We will recruit four consecutive freshman cohorts and conduct follow-up surveys during the study period. The major risk factors for smoking examined will include environmental, personal, cultural, and psychosocial risk factors. We will address our aims through both quantitative (longitudinal surveys) and qualitative (focus groups) methods, recruiting students from Haskell Indian Nations University. Each participant will be surveyed twice a year (once per semester). Findings from this study will be used to address the development of tailored cessation programs for smoking for AI/AN.

### Study participants

First year students at Haskell Indian Nations University (HINU) in Kansas will be recruited over four consecutive years and will be surveyed annually and repeatedly through year 5 of the study to examine key measures and determinants of initiation and use among this high risk group.

Our research team has skills in epidemiologic studies, qualitative research, longitudinal cohort studies, and in conducting collaborative studies with AI/AN.

### Study setting - Haskell Indian Nations University

This study will recruit students from HINU. The student population at HINU is diverse and unique. We chose this college because of the diversity of the student population, based on their representation of over 200 different tribes throughout the country. In addition, the investigators on this proposal have strong collaborative relationships with this institution, increasing the likelihood of achieving the specific aims of this proposal.

HINU is one of the oldest and well recognized AI/AN Universities in the US. Founded in 1884 as the US Industrial Training School, in Lawrence, Kansas, HINU was originally established to assimilate AI/AN children into mainstream America. The US wanted to solve the "Indian problem" and they viewed education as the fastest and most complete means of achieving that goal. Removing Indian children from their families and communities was believed to remove the influences preventing the AI/AN from becoming productive and acceptable members of the dominant society. HINU began with 15 students, ranging from grades 1 to 5, from across the country. By 1884, there were 606 students representing 36 states. HINU became a land grant institution in 1994 with a vision to become a national center for AI/AN education, research, fine arts, service and cultural programs that increase knowledge and support the educational needs of AI/AN.

Currently, HINU offers Baccalaureate of Arts and Science degrees in Elementary Education, American Indian Studies, Business Administration and Environmental Science. Associate Degrees are offered in Liberal Arts, Social Work, Theatre, Art, Pre-professional Education, Business Administration, Computer Information Systems, Entrepreneurial Studies and Tribal Management. Associate of Science degrees are offered in Natural Sciences and Resources. HINU has an average enrollment of over 1,000 AI/AN students each semester and offers innovative curricula oriented toward AI/AN cultures. HINU belongs to the following associations: the Consortium for Graduate Opportunities for American Indians, the American Indian Higher Education Consortium, the Higher Learning Commission, North Central Association of Colleges and Schools, the National Association of Intercollegiate Activities, American Indian College Fund and the American Council on Education. Approximately 300 new students enroll each year at HINU and are eligible to participate in this longitudinal study.

### Study design

We will use a longitudinal study design to recruit consecutive freshman cohorts of TCU students and follow them up over several years to examine the natural history of smoking. This line of research is crucial to address the tobacco related morbidity and mortality in this population. The use of a longitudinal survey design is preferable over a cross-sectional survey because we will be able to determine both transition rates and the factors that impact transition rates. We decided to use multiple follow-up time points to maximize our statistical analysis of the smoking behaviors in this population. In addition, longitudinal surveys will allow us to make conclusions beyond simple associations and correlations. We will also conduct focus groups with students from HINU to gather additional information related to smoking or non-smoking. Finally, because little is known about the metabolism of nicotine in this population, we will collect saliva samples each year from a sample of current smokers to examine the pharmacokinetics of nicotine metabolism. We present the inclusion and exclusion criteria for the TCU students in Table 1.

**Table 1:** Inclusion and exclusion criteria for the tribal college/university students

Inclusion Criteria	Exclusion Criteria
◆ Student at Haskell Indian Nations University ◆ Age at least 18 years or older ◆ Have a valid college/university email address, telephone number, and home address ◆ Willing to participate in all study components ◆ Willing to be followed-up over several years (1-4 years, depending on freshman cohort year)	◆ Planning to leave or graduate from the University before the end of the current academic year ◆ Unable to participate in all components of the study

### Implementation

The main purpose of this study is to document and understand changes in smoking behavior of AI/AN TCU students. The proposed research will be conducted in three phases as shown in Table 2. The project will be implemented over five years.

**Table 2:** Implementation plan

Stage 1: Development and Training	Hiring of staff, training for staff, establish CAB, develop and refine web-survey, pilot test web-survey, recruit participants for first cohort of freshman, conduct baseline surveys, conduct focus groups	Year 1
**Stage 2: Longitudinal Surveys of Consecutive Freshman Cohorts and Follow-up surveys**	Recruitment of students, conduct baseline surveys in fall and follow-ups in spring and fall, conduct focus groups (Year 2), collect saliva samples from current users	**Years 2 - 5**

**Stage 3: Data Analysis and Dissemination**	Continue recruitment and follow-up surveys, and focus groups, continue data analysis and management,	**Year 4**
	
	Presentations at professional conferences, preparation of manuscripts, preparation of intervention grant for smoking among tribal college students	**Year 5**

### Stage 1: development, training and focus groups

The objectives of this stage are to:

i. Develop and finalize survey instrument

ii. Develop and finalize web-site

iii. Pilot test the survey instruments

iv. Begin recruitment of students into study

v. Focus groups I

The first phase of the study will be devoted to developing survey instruments, developing the web-sites, developing and refining the survey, refining recruitment and retention plans and pilot testing of web-survey.

### Development of web-survey instrument

The projected content of the web-survey is described below. The final content will be informed from input from the Scientific Advisory Board (SAB) and co-investigators.

### Web-survey components

#### Demographics

Information such as year in school, age, gender, residence status (grew up on or near reservations), parental education, tribal background or representation, cowboy or rodeo status and others will be included in the survey. Results from meetings with the SAB, Community Advisory Boards (CAB) and co-investigators from our partnering organizations will provide additional variables and questions to include in the final longitudinal surveys.

#### Smoking behavior

Questions will include history of smoking, current smoking status, and susceptibility to smoking for never smokers. The key question categorizing college students into the main smoking categories is, "On how many of the past 30 days did you smoke cigarettes?" Participants who smoked on at least 1 day in the past month will be categorized as current smokers and those who report no use in the past month will be grouped into the non-user category. We will also ascertain for current users the average number of cigarettes smoked per day. We are aware that there are finer categories of use including never used, experimenter, and former user. We will use similar questions used in our previous research to measure different levels of uptake [21].

Participants will be asked questions related to the frequency of smoking per day, years of smoking, and age of initiation. They will also be asked the Smoking Dependence Scale.

Susceptibility to smoke will be assessed by using questions that were developed by members of this team in prior research. The questions will be modified, but to begin we will use: (1) Do you think you will try a cigarette soon? (2) If one of your best friends offered you a cigarette, would you use it? (3) Do you think you will be smoking a year from now? The response categories for these questions will range from "Definitely not, Probably not, Probably yes, and Definitely yes." As indicated from previous research, any response other than "Definitely not" to all three questions, will categorize the participant as susceptible to smoke and through this longitudinal design, we will be able to assess the validity of this measure for smoking among AI/AN TCU students.

#### Environmental factors

##### Household structure

Single parent households or households with the mother and a stepfather present are known to pose a risk for substance use [22]. A household structure measure will be created that will account for different types of households: both parents only, mother only, father only, mother and stepfather only, father and stepmother only, grandparents with or without others, and other.

##### Social support

Family caring and support have been identified as influential factors for substance use and tobacco use. We will use a social support scale that includes items from a measure developed by Zimet et al, consisting of six questions that asks respondents about the availability of friends, family, or a special person for emotional support and assistance (e.g., "My family really tries to help me," "I have a special person who is a real source of comfort to me," "I can talk about my problems with my friends"). A 5-point response format will be used for all these items, with choices ranging from "Disagree" to "Agree" (alpha = 0.84).

##### Exposure to smokers

Exposure to parental use as well as peer use is a risk factor for smoking. Therefore, we will create an exposure index from minimal (no family or friends use) to high (both familial and peer users) level of exposure to other smokers.

##### Tobacco marketing

Recent studies have shown that tobacco marketing affects smoking uptake and progression. We will ask questions related to tobacco advertising and promotions to assess level of exposure and receptiveness to these marketing campaigns [23]. Receptivity to tobacco marketing has been shown to influence behavior in the white college population, but no studies have demonstrated the influence of tobacco marketing on the uptake and smoking in AI/AN college students. This is especially important given the tobacco industry's marketing practices targeting college students and minority students.

#### Personal factors

##### Academic performance

Personal risk factors include academic performance at TCUs, which has also been shown to be a predictor of smoking behavior in this population. We will use self-reported academic performance with specific letter grades (A+ to F).

##### Social participation/community mindedness

Involvement in social and university activities has been shown to be a protective factor for tobacco use. We will collect information on each student's level of participation in sports, religion, student government, newspaper, as well as others. Having a constructive role in society has been found to be associated with less use. We will use a community mindedness scale which includes six questions that asks respondents how connected they are to relatives, elders, friends, and others in their communities (e.g., "I volunteer to help the elders," "How I act pleases my friends in the community"). All items use a 4-category response format with choices ranging from "Rarely or never" to "Almost always" (alpha = 0.87) [24].

##### AI/AN ethnic identity

The role of culture and ethnic identity is an important factor for AI/AN smokers [25,26]. Defining and measuring these concepts, however, is exceedingly difficult, particularly in this community. A few scales have been developed for acculturation and traditionalism and tested among AI/AN (e.g. - American Indian Acculturation Scale, Traditionalism Scale [27,28]); however, our pilot participants, community partners, and CAB members are not comfortable with any of them for several reasons. First, proposing to use any measure of acculturation in AI/AN presumes that they have been acculturated, which by definition is the *voluntary *blending of two or more cultures. The historic ethnocide and genocide of AI/AN people is an example of assimilation (the *forced *blending of cultures or forcing one group to accept the culture of another group) rather than acculturation. Therefore, measuring the degree to which AI/AN have become similar to the majority population is akin to measuring the success of colonialism and termination policies, not the blending of cultures themselves. Second, acculturation scales (and traditionalism scales) tend to use measures such as language use and participation in "cultural" activities. Once again, they are invalid in this community as the majority of Native languages and practices were forcibly stopped through boarding schools, the Courts of Indian Offenses, and termination policies. It is impossible to measure an individual's ties to his or her culture by measuring things that have been ended by an act of law. Third, the heterogeneity of the AI/AN population would require over 500 different scales to measure ties to different cultures. By making the assumption that there is one "identity" for all AI/AN people we essentially measure the culture of one group, thus negating others. For example, if participating in ceremonial use of tobacco is a tie to one's ethnic identity, then AI/AN people who do not use tobacco in that way would be less "Indian". As traditional use of tobacco is incredible heterogeneous, we would be calling groups like most Alaska Natives less "Indian". Finally, though ethnic identity scales have met with more acceptance among our community members, they are still problematic as they attempt to force a more concrete definition onto ethnic identity, which is an abstract concept. During phase I of this project we will work with our community partners and CAB members to attempt to create a scale that makes sense for what we are trying to measure. If a suitable scale cannot be created, we will use an open-ended question to ask people how they understand their ethnic identity or cultural heritage.

#### Psychosocial Factors

##### Depressive symptoms

Previous studies show that depressive symptoms are predictive of tobacco use in college students [29]. We will use a validated scale of depressive symptoms to assess this in tribal college students [29]. The internal consistency of the depressive symptom scale was 0.72 as measured by the Cronbach's coefficient alpha statistic. The specific items for this measure are: "During the past year, how often have you felt...1) too tired to do things? 2) had trouble going to sleep or staying asleep? 3) felt unhappy, sad or depressed? 4) felt hopeless about the future? 5) felt nervous or tense and 6) worried too much about things?" The response categories for each question are: "Often, Sometimes, Rarely or Never." These four responses are assigned scores of 1, 2, 3 and 4, respectively, and then summed to produce an overall depressive symptom score, ranging from 6 to 24 points. Respondents will be categorized as having or not having depressive symptoms based on a previously validated cut-off score[29]. Due to higher levels of stress and adjusting to college life, students may experience more depressive symptoms.

AI/AN adults are thought to experience significant depressive symptoms at rates several times higher than other adults, yet we know very little about factors associated with depressive symptoms among this under-studied group. Many researchers argue that depressive symptoms are associated with conflicts between AI/AN traditional cultural values, practices, and beliefs and those of the majority culture. A recent study based on a sample of 287 AI/AN adults from the upper Midwest takes into account two measures of cultural effects: perceived discrimination, as one indicator of culture conflict, and traditional practices, as a measure of cultural identification [30]. The results indicate that discrimination is strongly associated with depressive symptoms among AI/AN adults and that engaging in traditional practices is negatively related to depressive symptoms. Interaction effects between perceived discrimination and traditional practices indicate that engaging in traditional practices buffers the negative effects of discrimination among those who regularly participate in them.

### Timing of follow-up surveys

Table 3 shows the major areas of each section of the survey and the timing of data collection. We identify other areas by major categories. Again, these items are validated items and have sound psychometric properties, and they have been used previously in national surveys [31]. However, because many of the measures listed above have not been validated with AI/AN, in the current study we will explore the psychometric properties of these measures (e.g., internal consistency - Cronbach's alpha). Students will be surveyed in the fall and followed-up at the end of the spring semester. Additional questions and areas of interest will be added to the longitudinal student surveys.

**Table 3:** Primary questions in prevalence and longitudinal student surveys and the timing and frequency of follow-ups for the longitudinal surveys

Student Surveys	Baseline Survey (Fall)	Follow-up Surveys (Spring)
**Demographic **(age, gender, parental education, year in school, etc)	X	

**Environmental factors**		

Household structure	X	X

Social support	X	X

Exposure to other smokers	X	X

Exposure to tobacco marketing	X	X

**Psychosocial factors**		

Depressive symptoms	X	X

**Personal factors**		

Academic achievement	X	X

Rebelliousness	X	X

Social participation	X	X

**Ethnic Identity **(American Indian/Alaska Native)	X	X

**Smoking behavior**		

Ceremonial/Non-ceremonial uses of tobacco	X	X

Type of cigarettes (menthol vs. non-menthol)		

Smoking history/current smoking status	X	X

Susceptibility to smoking status	X	X

Smoking Dependence Scale	X	X

Cigarettes per day, duration of smoking		

Quit attempts/quitting history	X	X

**Other factors**		

Other forms of tobacco use (smoking)	X	X

Alcohol intake (amount, binge drinking, etc)	X	X

Tobacco policy/rule in dormitory/living place	X	X

**Nicotine metabolism**		
Saliva samples (sub-sample of current users)	X	X

### Web-based surveys (programming and development)

#### Comprehensive Research Information System (CRIS)

This study will use a state-of-the-art Clinical Information Management Database System. It is a secure, 21 CFR Part 11-compliant, robust and scalable system such that data and protocol information can be entered efficiently and in a standardized format. This web-based system allows for direct data entry from participating institutions. The comprehensive database management system supports participant recruitment, study monitoring, trial design, protocol management and data safety monitoring; case report form construction and dissemination; integration of tissue and clinical information; clinical trial execution and query management; and integration with third party clinical systems.

Our CRIS provides capabilities to: 1) create, maintain and edit participant data, such as demographics, labs, medications, diagnosis, clinical history and tissue samples; 2) design and develop research protocols; 3) track the development of study protocols, versions, amendments and IRB approvals/renewals; 4) create participant screening and enrolling criteria; 5) create and disseminate case report forms for clinical trials and outcomes studies; 6) create and maintain tissue banks and associate tissue samples with other clinical data at both participant and study levels; 7) create participant schedules and record clinical results and participant status in research protocols; 8) create user and multi-organization research networks; 9) record, maintain and report adverse events; 10) store and report on all participant- and study-level clinical data; 11) conduct study queries and generate standard and ad hoc clinical reports; 12) export clinical data to third party analytical tools, such as SAS and Excel. In addition, the system will help with the coordination of enrollment for multicenter studies, increase oversight of participating centers, allow for real-time monitoring of enrollment and patient data with built-in data quality, auditing and integrity checks. The system provides our researchers with a central repository for all study related documents and allows for automation of research administration activities thereby reducing time to study activation.

The comprehensive research information system is HL-7 compliant that is configured to easily integrate with internal as well as third-party lab systems, Electronic Medical Record systems, etc., through one integrally-designed system. The system supports multi-center, cooperative group and investigator-initiated research through advanced technology and security features, all contained in one comprehensive environment. Through utilization of this comprehensive system great improvements in the research productivity, efficiency, collaboration and integrity of data can be achieved.

#### Tribal Student Portal

The Tribal Student Portal is a separate website from the CRIS database that will be linked to our culturally tailored website about tobacco and health. The Tribal Student Portal website is linked with the CRIS database so that any form or survey completed within the Tribal Student Portal is transmitted back to the CRIS database and resides there under that specific patient information. This is how we will protect the integrity and security of the data collected. The students will not have access to the actual CRIS database and they cannot alter the form or survey housed on the Tribal Student Portal website, they can only fill it out and submit it.

#### Data collection form development

Through previous collaborations with various clinical research groups, our Center for Biostatistics and Advanced Informatics (CBAI) staff has over 40 years collective experience in the design, development, validation and implementation of data collection forms that are consistent with protocol, reporting and various sponsor requirements. The design of the data collection forms will be collaboration between the Clinical Information Specialists of the CBAI, the project manager, the Principal Investigator, and the study statistician. Once all variables of interest have been identified and verified with the study endpoints and proposed analysis the initial drafts of the forms will be designed in the development environment of the CRIS. Once the draft forms are created, they will be reviewed for completeness, accuracy and utilization by coordinators from the various sites. Mock data collection forms will be completed to ensure compatibility with the data being collected in the source documentation as well as used for validation of the range, logic and other edit checks in the web-based data entry system. Once forms are validated and finalized, they will be locked to format and editing in the system and added to the production environment for coordinators to remotely enter data.

Students who respond to our recruitment email expressing interest in participating in the study will be assigned a unique identifier upon their first visit to the site. This ID will allow students to save surveys in progress and return later for completion. This will also allow the students to return later for additional surveys. Each student will be emailed directions for the location of the web-survey, once they are at the website, they will have to enter their unique ID and a password, to access the surveys.

### Web-survey technical difficulties

Using a web application will ensure that all students will be able to access the survey. The minimum requirements will be a computer running an Internet Explorer version 4.0+ or equivalent and access to the internet. This includes more than 99% of all computers (browser usage from http://www.thecounter.com/stats/). Students who forget or lose their unique ID and password will be addressed by having users identify and answer "secret" questions upon their first visit to the site (Ex. First pets name?). This information will be stored separate from study data. A student who forgets his/her ID will be able to retrieve it by answering the questions they identified and answered.

### Pilot testing of web-surveys

We will use a web-based survey design for all students. We will pilot the web-survey to a sample of 25 students. Since data collection is almost instantaneous, we will be able to analyze the questions for logistical problems such as skip patterns, range errors, and response issues. For this pilot testing of the web-surveys, we will add a comment box at the end of the survey for feedback from the students with respect to the survey components and any additional comments related to recruitment and retention for the longitudinal study.

After completing the web survey (i.e., baseline) students will be asked: 1) if they felt the survey questions clearly assessed the important aspects of smoking uptake and cessation, issues related to traditional/ceremonial use of tobacco, 2) whether they felt comfortable responding to all of the questions, 3) their perception of the clarity and completeness of the information included in the web survey, 4) the personal relevancy of the survey topics and 5) the readability/presentation of the web survey. Survey items will be revised based on feedback.

### Focus groups I

We will conduct focus groups in year 1 of the study to understand AI/AN college students' knowledge, attitudes and beliefs about smoking and its relationship to smoking and traditional or ceremonial use of tobacco. We plan to recruit college freshman and conduct the focus groups during the first semester of college to learn about students' views early in their college careers. The surveys given in later years will be based on these results to better understand changing beliefs over the course of the 4 years of college.

#### Sampling frame

We will use a stratified, nested sampling frame, with divisions based on gender and smoking. We stratify by gender because, based on our experience with the AI/AN population, college men and women are more likely to speak openly when divided by gender. We stratify by smoking because we believe there will be substantial differences in knowledge, attitudes and beliefs based on use status. We plan for 3 focus groups per stratum, but will assess saturation after conducting two focus groups per stratum and will cease recruitment if our data are saturated. In addition, it may not be possible to complete 3 groups of 10 students in each stratum, depending on the number of entering students and the prevalence of smoking in that particular class. We will stop recruitment if we determine we have reached all possible willing participants. We plan a focus group of 10 students per group as shown in Table 4.

**Table 4:** Organization of focus group I

Focus Group I			
**Men**		**Women**	

**Smoker**	**Non-smoker**	**Smoker**	**Non-smoker**

3	3	3	3

N = 30	N = 30	N = 30	N = 30

Total: 12 groups (120 participants)			

#### Recruitment

Students will be recruited for focus groups through word of mouth and flyers or posters at each campus. We will also make announcements at orientation to recruit as we have found it to be an efficient way to recruit for focus groups. Using these techniques we have been able to recruit 109 HINU students for focus groups in less than 6 weeks. We conservatively plan for 3 months of recruitment for these groups, which will be conducted during the fall semester.

#### Development of Focus Group Moderator's Guide

We will develop our focus group moderator's guide during the first few months of the study, based on guides examining knowledge, attitudes and beliefs about smoking among AI/AN. Guides will be modified for each stratum as appropriate. We will get input from our CAB and SAB members for additional areas to discuss. In general, we will cover the following topics: knowledge of what smoking is and who among their peers use it, reasons for smoking (either personal or peers), when and why people start using it (including peer pressure, liking the way it feels or looks, "rebellion" from parents, emulating parents or other influential people, etc.), if they or anyone they know has ever quit, how and why people they know have quit, how smoking is similar or different from using smokeless tobacco, how smoking is similar or different from the use of traditional tobacco and the health consequences of smoking.

#### Focus Group Procedures

Focus groups, lasting approximately 60 to 90 minutes, will be conducted on campus. Groups will be conducted at a variety of times to allow for participation of individuals with different schedules. Focus groups will be audio-taped and transcribed verbatim. Focus groups will be moderated by gender- and ethnically-matched research assistants that have been trained to conduct focus groups. We have found that matching gender and ethnicity when selecting focus group moderators is imperative to gaining richly detailed information, particularly among younger students. Prior to the start of the focus group, all students will provide both written and verbal informed consent. All students will receive a $25 gift card for their time and participation in the focus group.

#### Focus Group Analysis

All verbatim transcripts will be entered into the nVivo program [32], a software program designed for cataloging and analyzing qualitative data. Text analysis will follow a grounded theory [33] approach and will use techniques developed by Dr. Daley during analysis of qualitative needs assessment data from work in the Kansas City AI/AN community. In grounded theory, categories and concepts emerge from the text and are then linked to formal theories. Using this approach will allow us to develop a more accurate theoretical framework and model of behavior. Analysis techniques follow a series of steps based in Community Based Participatory Research (CBPR) and ethnography as shown in Table 5. Analysts include the following:

**Table 5:** Focus group analysis plan

Role	Qualifications	Responsibilities
*Primary Coder*	• Formally trained in qualitative methods • Member of the research team who is not a community member • Did not take part in the focus groups	• Leads coding meetings • Responsible for code book upkeep • Responsible for formal drafting of initial themes and sub-themes • Participates in all coder activities described below

*Secondary Coder*	• Formally trained in qualitative methods • Member of the research team who is also a community member • Lead at least some of the focus groups	• Responsible for identification of representative quotes • Responsible for review of themes and sub-themes prior to sending to reviewers • Participates in all coder activities described below

*Tertiary Coder*	• Formally trained in qualitative methods • Member of the research team (can be a community member or not) • May or may not have taken part in the focus groups	• Participates in all coder activities described below

*Emic Reviewer*	• Formally trained in qualitative methods • Member of the research team who is a community member • Did not take part in the focus groups	• Makes final determination on representative quotes • Works with etic reviewer to finalize all themes and sub-themes

*Etic Reviewer*	• Formally trained in qualitative methods • Member of the research team who is not a community member • Did not take part in the focus groups	• Leads overall analysis • Works with emic reviewer to finalize all themes and sub-themes

Analytic coders first read through the transcripts and inductively come up with topic and sub-topic areas. The coders then meet and develop an initial code book based on these topics. Coders then reread transcripts, beginning deductive coding with the code book, but also including any inductive coding necessary to complete topic and subtopic areas. Coders meet again to finalize the code book and then go back and reread the transcripts, completing all deductive coding. Coders meet a third time to discuss themes present in the topic areas, coming up with basic ideas about thematic statements, which are then drafted by the primary coder and sent to the secondary coder for review. Coders meet a final time to discuss and come to consensus on the thematic statements before they are finalized by the primary coder and sent to the emic and etic reviewers. The reviewers discuss the themes together and come up with finalized statements which are brought back to the entire analytic team for consensus. Following this process using the roles defined above, allows the research team to ensure that both the etic or "objective and scientific" point of view that is often sought in qualitative research comes through, as well as the emic or "insider"/community view, which is necessary for CBPR. The thematic statements can then be developed into any needed theories using grounded theory where the data are allowed to form their own theory rather than being placed into an existing theory. Each stratum will be analyzed separately prior to comparison among strata to ensure data are not contaminated.

### Stage 2: Longitudinal surveys and focus groups II

The objectives of this stage are to:

i. Recruit students from HINU

ii. Conduct a second set of focus groups to understand potential smoking interventions

iii. Conduct follow-up surveys of students

iv. Collect saliva samples from current smokers

### Recruitment of participants

Recruitment for the longitudinal surveys will occur using the following methods: 1) electronic invitation via university email and 2) research staff and faculty on campus and at the student centers. Recruitment posters and flyers will be posted and mailers will also be sent out inviting participation by eligible participants. Table 6 shows our expected participant accrual.

**Table 6:** Participants accrual

	Year in College				
**Cohort Year**	**Freshman**	**Sophomore**	**Junior**	**Senior**	**TOTAL**

2009 - 10	250*				250

2010 - 11	250	137**			387

2011 - 12	**250**	137	96***		483

2012 - 13	250	**137**	96	**67**	550

2013 - 14	**250**	137	**96**	67	550

**TOTAL**					**2,220**

* Based on our previous work with the tribal college students, we estimate that we will be able to successfully recruit 85% of the freshman cohort each year of this project.

** Tribal college students have a high dropout rate from freshman to sophomore year; we assume a 45% drop out rate.

*** The dropout rate reduces in subsequent years, so we assume a 30% drop out rate in each subsequent year.

### Email invitation to college sample

The primary recruitment strategy will be by email announcements on campus. We will attempt to recruit the entire freshman class each year since the number of first year students is manageable. This email will be sent out by a designated official of HINU. The emails will contain information and instructions for the study and the website that contains the survey instrument. The Tribal Student Portal feature includes an eConsent that must be accepted prior to filling out the survey. Just like the longitudinal survey, the eConsent is a form that will be transmitted back to the CRIS database and housed under that student's information. Because the ID and password are unique and directly connected to a student, acceptance is considered an electronic signature. When the student logs into the Tribal Student Portal website, the eConsent will appear. Before the student can proceed with completing forms or surveys they have to complete the eConsent and submit it. After they submit the eConsent, the survey will appear for the student to complete and proceed with the study.

### Research staff and faculty

Information describing the longitudinal cohort study will be announced as well as flyers passed out at the campus. In addition, the CAB members along with the investigators will make presentations on campus at student activities and forums. Recruitment and retention of college students in this study are issues of utmost importance for the study's success. According to our sample size calculations, even if we assume a modest level of participation, we will have sufficient number of eligible students for this study. Progress in recruitment will be continuously evaluated by the PI, co-investigators and SAB. Input from the team will be elicited to enhance recruitment and retention strategies.

### Focus groups II

We plan a second set of focus groups in year two. These groups will focus on possible interventions to help college students quit smoking. Because we are looking for more specific information, our sampling frame will be modified as presented in Table 7, dividing students based on current versus prior use of cigarettes. Participants from the previous set of focus groups will not be excluded because the topics to be discussed are different. We will use the same recruitment strategies for these groups as were used for our first set of focus groups. Focus group procedures and analysis will follow the same approach as our first set of focus groups. We plan to cover the following general topics, though modifications will be made based on our first set of groups and comments from our SAB and CAB: reasons for use and initiation of smoking, reasons for quitting (if appropriate), descriptions of prior quit attempts and why they succeeded or failed, types of things they would like to see in an intervention, types of interventions (e.g. - in person, on-line, group vs. individual, etc.) and if and how traditional tobacco should be discussed in a smoking cessation program. Information from these focus groups will help us to determine appropriate intervention strategies for AI/AN college students.

**Table 7:** Organization of focus group II

Focus Group II			
**Men**		**Women**	

**Smoker**	**Former smoker**	**Smoker**	**Former smoker**

3	3	3	3

N = 30	N = 30	N = 30	N = 30

Total: 12 groups (120 participants)			

### Maintenance of student cohort over the study period

#### Previous studies and retention rates

We have conducted several studies among AI/AN participants. We were able to achieve over 95% participation rates of freshman at HINU to participate in a cross-sectional survey of health behaviors.

#### Strategies to enhance response rates

To maximize the likelihood that selected students will complete the survey, the research team will conduct a number of activities before and after sending the invitation to selected students. In addition, Advisory Board members will review the recruitment and retention plan and suggest additional strategies that may be college-specific (e.g., ads in college newspapers). Planned activities are outlined below. Students who participate in the baseline and follow-up surveys will be given a $10 gift card and the same amount will be provided for those who complete each follow-up survey.

#### Follow-up activities

We will conduct three follow-up activities with those who do not respond to the first invitation. First, we will send reminders (emails, letters or postcards). Second, research assistants will leave a phone message for students to complete their surveys online. Third, we will contact non-respondents and offer to complete the survey on the phone. We will make up to 5 telephone calls for each participant. We anticipate that these extensive efforts will yield target response rates necessary to carry out this study.

### Quality assurance

All research staff will be trained in a standardized manner. Each team member will receive 2 hours of recruitment training, which will include: 1) A detailed discussion of study objectives and responsibilities for facilitating student recruitment; 2) a timeline for recruitment related tasks; 3) scripts for recruitment; 4) role-plays of potential recruitment scenarios and 5) problem-solving around difficult recruitment situations. For the web-based longitudinal survey, as the information is being collected at the University of Kansas Medical Center (KUMC), we will conduct random checks of the data collection. We will use procedures that have been used by the investigators in the previous projects. The PI will monitor the data on a monthly basis to ensure quality control of the data. In addition, the PI will meet with the research staff on a weekly basis for supervision, to troubleshoot problem with the data collection and to reinforce staff compliance with the protocol.

### Collection of saliva from a sample population of current smokers

Each year, we will recruit a sample of current smokers from HINU to provide saliva samples to examine the pharmacokinetics of nicotine metabolism among this population. One of the questions on the Tribal Student Portal website survey will ask each participant if they would be willing to provide saliva samples in person (if they are a current smokers). These students will be contacted and if they agree to provide saliva samples, our research assistants will collect the samples and send them back to our respective study site for storage in a freezer until shipment to the laboratory for analysis. Since cotinine in saliva is very stable, mailing the samples via overnight mail prior to freezing is not a concern.

### Stage 3: data analysis

The beginning of year 5 of the study will be devoted to data analysis and report writing. For students who start the study and either drop out or miss any of the scheduled surveys, we will attempt to obtain the main outcome measures (smoking status) via the telephone or mail or email.

Prior to the main analyses, the distributions of all variables will be inspected and transformed, if necessary, to ensure that they meet the assumptions of the statistical tests employed. Initial analyses will be conducted to evaluate the baseline distribution of the main smoking status variables as well as the other risk factors for smoking.

### Analysis of Study Aim 1: To examine the natural history of Smoking among AI/AN TCU students and examine risk factors

a) To estimate the prevalence of smoking in this population.

Results of the baseline survey will be used to estimate the prevalence of smoking among AI/AN college students. The smoking status at baseline for each of the n individuals surveyed is a dichotomous random variable such that, for the ith individual, Y_i _= {1 if smoker; 0 if non-smoker}. (See the Precision Analysis section below for a discussion of the total sample size [n].) Summing these n independent random variables will yield the random variable that follows a Binomial(n,π) distribution, where π represents the smoking prevalence for this population. The maximum likelihood estimate (MLE) for π is $\bar{\text{Y}}$ (the average of the Y_i_'s), and the variance of this estimate can be approximated by the formula $\bar{\text{Y}}(1-\bar{\text{Y}})/\text{n}$ (Hogg RV, Craig AT, 1995). We will use these values to estimate the prevalence and generate a 95% confidence interval (CI) for this estimate.

b) To determine the rates of smoking uptake (non-use to experimentation to established use) in this population.

Students self-identifying as non-users in the baseline survey will be used to estimate the smoking uptake rate in this population. The smoking status at follow-up will be assessed for these n_non _students. (See the Precision Analysis section below for a discussion of the sample size of non-users [n_non_].) This yields a dichotomous random variable such that, for the ith individual, Y_i _= {1 if became user; 0 if remained non-user}. Again employing the binomial distribution, we will estimate smoking uptake rate and generate a 95% CI for this estimate using the methods described in Study Aim 2, part a (above), substituting n_non _into the formulation in place of n.

In addition to the analyses described in the paragraph above, we will also follow smoking status over our multiple follow-up time points. This will be done two different ways: one using a dichotomous outcome variable (smoker/non-smoker) at each of the time points and the other using three smoking stages (non-user/experimental user/established user) over the different time points. For this first, we will use generalized estimating equation[34] and Generalized Linear Mixed Model methodologies[35]. These established approaches have been incorporated into mainstream statistical software (e.g., SAS PROC GENMOD for GEE and SAS PROC NLMIXED for GLMM). This analysis will allow us to examine smoking prevalence longitudinally. We will compare and contrast our results from these two methods, as well as assess the model assumptions to determine the most appropriate method for our data. The second type of analysis considers three smoking stages (non-user/experimental user/established user) over the follow-up time points. For this analysis, we will examine the transition probabilities (e.g., from non-user to experimental, from established to non-user, etc.) using a discrete time Markov chain[36,37]. The Markov chain approach is well suited to study outcomes such as these due to the discrete stages and the repeated measures over time.

c) To determine the rates of smoking cessation in this population.

Students self-identifying as smokers in the baseline survey will be used to estimate the smoking cessation rate in this population. The smoking status at follow-up will be assessed for these n_smk _subjects. This yields a dichotomous random variable such that, for the ith individual, Y_i _= {1 if quit; 0 if remained smoker}. (See the Precision Analysis section below for a discussion of the sample size of smokers.) Again employing the binomial distribution, we will estimate smoking cessation rate and generate a 95% CI for this estimate using the methods described in Study Aim 2, part a (above), substituting n_smk _into the formulation in place of n.

### Analysis of Study Aim 2: To examine the factors associated with smoking and cessation (e.g., tobacco marketing, traditional tobacco use, cultural identification) among AI/AN TCU students

Students self-identifying as non-users in the baseline survey will be used to examine the predictors of smoking uptake in this population. The smoking status at follow-up will be assessed for these participants. This yields a dichotomous random variable such that, for the ith individual, Y_i _= {1 if became user; 0 if remained non-user}. The random variable Y_i _is assumed to follow a Bernoulli distribution with a mean parameter π_i_; so E[Y_i_] = π_i_. Unconditional logistic regression techniques can then be used to model the function of the mean logit[π_i_] = ln[π_i_/(1 - π_i_)] = η_i _= x_i_'β. In this model, x_i_' = (1, x'_demog_, x'_traditional use_, x'_environ., _+ others) is the set of predictor variables for the ith observation. The vector β = (β_intercept_, β'_demog_, β'_traditional use_, β'_environ, + _others)' represents the values of logit[π_i_] for each 1-unit increase in the correspondingly subscripted value from x_i_'. Wald χ^2 ^tests will be used to test for significant differences in smoking uptake between various subgroups of AI/AN students (Hosmer DW, Lemeshow S, 2000). The Deviance statistic and the Hosmer-Lemeshow goodness-of-fit test will be used to assess the fit of the models (Hosmer DW, Lemeshow S, 2000). The MLE of π_i _(say ${\overset{\wedge }{\pi }}_{i}$) can also be calculated by transforming the MLE of logit[π_i_] (say log∧it[πi]=x¯i'β¯∧) (Hogg RV, Craig AT, 1995). In other words, using logistic regression, we will be able to generate estimates for smoking uptake within different subgroups of our sample. These estimates can also be adjusted for other covariates, such as demographic characteristics.

Subjects self-identifying as smokers in the baseline survey will be used to examine the predictors of smoking cessation in this population. The smoking status at follow-up will be assessed for these subjects. This yields a dichotomous random variable such that, for the ith individual, Y_i _= {1 if quit smoking; 0 if remained user}. The unconditional logistic regression methods to examine variables that predict smoking cessation will be the same as those described above to predict uptake.

### Analysis of Study Aim 3: To examine the nicotine metabolism among current smokers in the AI/AN tribal college student population

Concentrations of nicotine and cotinine will be determined by gas chromatography (GC) with nitrogen-phosphorus detection. Frozen saliva samples will be shipped to the laboratory for detailed analysis as described above.

### Sample size calculations and precision analysis for longitudinal survey

There are four major analyses in this proposal: (1) estimate the prevalence of smoking in this population; (2) determine the rates of smoking uptake (non-use to experimentation to established use), (3) determine the rates of smoking cessation and (4) examine the factors associated with smoker and cessation (e.g., tobacco marketing, traditional tobacco use, cultural identification) among AI/AN TCU students.

For analyses (1) - (3) the sample size selected will reduce the size of the 95% CIs around the smoking uptake and prevalence rate estimates to within ± 18.9% of their respective point estimates. Using the formula $\bar{\text{Y}}(1-\bar{\text{Y}})/\text{n}$ to estimate the variance (Hogg RV, Craig AT, 1995) of the smoking uptake rate, the 95% CI will be within ±$1.96\sqrt{\bar{\text{Y}}(1-\bar{\text{Y}})/\text{n}}$ of the point estimate for uptake rate (called margin of error). The most conservative estimate is to assume that $\bar{\text{Y}}$ = 0.5. Therefore, the margin of error is $1.96\sqrt{\bar{\text{Y}}(1-\bar{\text{Y}})/\text{n}}$ = 0.98/√n. The sample size n depends on the analysis. For analysis (1) we have five cohorts each with 250 participants = 5*250 = 1250 which leads to a margin of error of 2.8%. Since we have attrition and different lags for cohorts, the analysis for the smoking uptake and cessation requires further explanation using a conservative argument. The least amount of subjects will be in the senior year (67*2 = 134). Assuming 20% smoking rate (80% non-smoker) at baseline (n = 134*.8 = 107) we have a margin of error for smoking uptake across four years to be 9.5%. Both 2.8% and 9.5% are acceptable margin of errors. We will have higher margin of error for estimating smoking and cessation (n = 134*.2 = 27) for a margin of error of 18.9% but will be smaller for cessation at years 1 and 2 follow-up.

Using the same argument, we will have at minimum 134 participants across four years (more for intermediate years) that can be used to determine factors associated with smoking use and 27 for smoking cessation. This will allow over 13 predictors (10 variables per predictor) in a logistic regression model across four years for use and about 3 predictors for cessation. In summary, even with conservative calculations in the previous two paragraphs, we have adequate sample sizes to accomplish the analysis to support the aims of this study.

### Dissemination

The results of this highly innovative longitudinal cohort study should provide effective dissemination opportunities. As with all of our research, a first-level dissemination of results will take place to local stakeholders, including our participant TCU, community members, the local IHS Service Units, Tribal Health departments, Tribal Councils and local media. Next, we will disseminate nationally to 3 audiences: first, the overarching American Indian Higher Education Consortium, comprised of all 39 TCUs; second, the IHS national leadership during their annual CASUD (Council of Clinical and Service Unit Directors) meeting, with the goal of educating administrators and clinicians about smoking; third, the annual gathering of all the Tribal self-governance and compacting tribes (i.e., those that have taken over control for their health services). They are also a ripe potential audience for learning about our results. Beyond these, other possible venues for dissemination with sustainability implications include the National Congress of American Indians, which has an active Health Caucus, and the National Indian Gaming Association.

### Strengths of this study

#### AI/AN TCU population

Few studies have examined smoking among AI/AN college students and we are aware of no longitudinal studies. This is important because the smoking rate in this population is the highest among any ethnic group in the US. The tobacco attributable morbidity and mortality is also extremely high in this population and reducing the long term smoking rates will contribute to lowering the health hazards related to smoking.

#### Nicotine metabolism

We will be collecting saliva samples from current smokers to estimate the nicotine metabolism in this population. This aspect of the study is important for future pharmacologic implications related to cessation programs for smokers.

#### Longitudinal Cohort Study

The use of a multi-wave longitudinal cohort design will allow us to examine transitions and risk factors for progression over time so that we will be able to assess the different predictors of smoking among college students. In addition, we plan to recruit consecutive freshman cohorts to assess trends over time taking into consideration any cohort effects. Examining natural progression is not always possible with an intervention design and we feel that additional research would be helpful before developing an evidence-based, randomized intervention trial. The longitudinal design is much stronger than a cross-sectional survey, where only associations and correlates can be assessed.

## Competing interests

The authors declare that they have no competing interests.

## Authors' contributions

BF, CMD, BG, and WSC participated in the design of the study; CMP, BF, CMD, and WSC contributed to the coordination of the study; BG led the statistical and power calculations. All the authors have read, revised and approved the final manuscript.

## Pre-publication history

The pre-publication history for this paper can be accessed here:

http://www.biomedcentral.com/1471-2458/10/617/prepub
